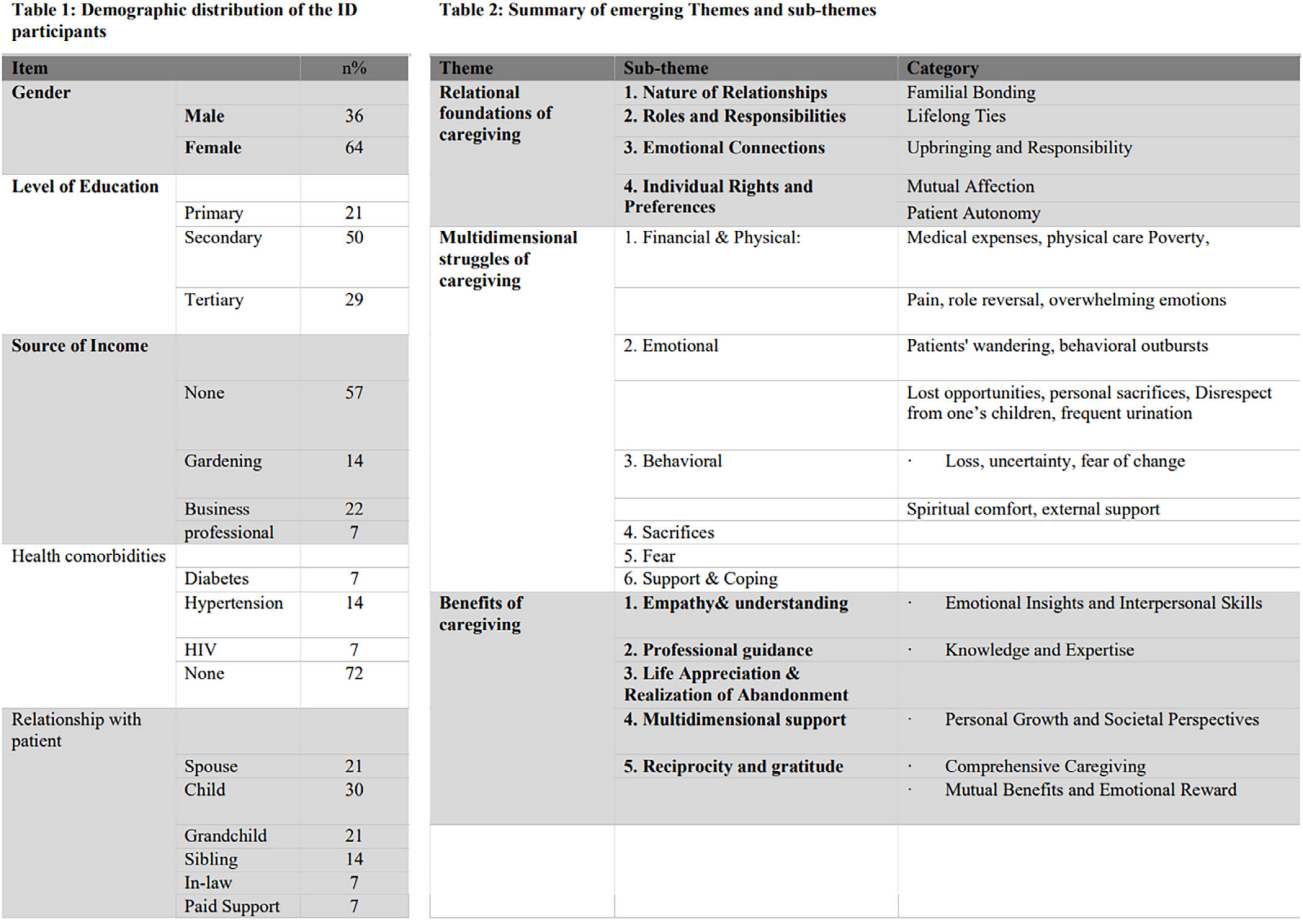# Understanding the Complexities of Caregiving for Alzheimer’s Disease and Related Dementias in Uganda: A Qualitative Insight

**DOI:** 10.1002/alz.084899

**Published:** 2025-01-09

**Authors:** Joy Onoria

**Affiliations:** ^1^ 7072 mulago hill, Kampala Uganda

## Abstract

**Background:**

In Uganda, caregivers of Alzheimer’s disease and related dementias (ADRD) face complex challenges, often lacking specialized skills for effective caregiving. Despite the growing prevalence of ADRD in the country, there’s a significant literature gap on caregiver selection and obstacles faced in urban and rural areas. This study aims to provide insights into the experiences of Ugandan caregivers, enhancing understanding of ADRD caregiving.

**Method:**

This qualitative study involved 14 caregivers from Nansana and Busukuma, suburban areas of Wakiso District, Uganda. A purposive sampling strategy ensured representation across diverse age and sex groups. In‐depth interviews, each lasting to 60‐90 minutes, were conducted in Luganda to ensure cultural relevance. The interviews were meticulously transcribed verbatim, forming the basis of thematic analysis. This analysis involved a detailed coding process to compare and examine participants’ responses regarding uniformity, emerging patterns, and contradictions, thereby offering nuanced insights into the caregiving experience in ADRD.

**Result:**

The study identified three primary themes within the caregiving experience for ADRD: relational foundations, multidimensional struggles, and the positive outcomes of caregiving. Caregiver selection is primarily influenced by existing family dynamics, with emotional bonds and patient preferences as crucial factors. Caregivers reported experiencing a spectrum of challenges, including emotional tolls, physical demands, and considerable financial sacrifices such as role reversals and the physical burden of care. Despite these challenges, many caregivers identified positive aspects, including enhanced empathy, acquisition of professional caregiving skills, and deepened sense of reciprocity. The thematic analysis provided a nuanced view of these complexities and benefits.

**Conclusion:**

This study offers vital insights into the experiences of caregivers of patients with ADRD in Uganda, highlighting the need for culturally sensitive support systems that integrate familial dynamics and caregiver experiences into healthcare policies. Recommendations include tailored training, community‐based networks, and digital platforms for remote support. Future research should broaden caregiver demographics and employ longitudinal studies. These findings are crucial for enhancing caregivers’ and patients’ quality of life, emphasizing the indispensable role of caregivers in ADRD management in Uganda and similar settings.